# Genome Sequence of the Plant Endophyte *Bacillus pumilus* INR7, Triggering Induced Systemic Resistance in Field Crops

**DOI:** 10.1128/genomeA.01093-14

**Published:** 2014-10-30

**Authors:** Haeyoung Jeong, Soo-Keun Choi, Joseph W. Kloepper, Choong-Min Ryu

**Affiliations:** a Korean Bioinformation Center, Korea Research Institute of Bioscience and Biotechnology (KRIBB), Daejeon, Republic of Korea; b Super-Bacteria Research Center, KRIBB, Daejeon, Republic of Korea; c Department of Entomology and Plant Pathology, Auburn University, Auburn, Alabama, USA

## Abstract

*Bacillus pumilus* INR7 is an endophytic bacterium that has been commercialized as a biological control product against soilborne pathogens as well as foliar pathogens by direct antagonism and induction of systemic resistance. In the current study, we provide the genome sequence and a possible explanation of the function of strain INR7.

## GENOME ANNOUNCEMENT

*Bacillus pumilus* INR7 was originally isolated from a surface-sterilized stem of a surviving cucumber plant in a field heavily infested with cucurbit wilt disease caused by *Erwinia tracheiphila* (1). Initially, application with strain INR7 on the seeds triggered plant systemic defenses referred to as induced systemic resistance (ISR) against several plant pathogens, including *Pseudomonas syringae* pv. lachrymans, *Colletotrichum orbiculare*, Cucumber mosaic virus, and *Ralstonia solanacearum*, and increased marketable cucumber yield (2, 3). In addition, the severity of cucurbit wilt and the numbers of cucumber beetles feeding on cucumbers were reduced following seed treatment with strain INR7 (4). In a separate study, an additive effect on the level of disease protection was reported following inoculation of a combination treatment composed of strain INR7 with a chemical trigger, benzothiadiazole (BTH), in a pepper field (5).

The strain INR7, designated Yield Shield and produced by Bayer Crop Science, is a unique case for a biocontrol product that has been developed using the capacity of a single entophytic bacilli to elicit both ISR and plant growth promotion. The product received registration from the United States Environmental Protection Agency (EPA) in 2003 for use on soybeans to protect against damping-off pathogens *Rhizoctonia solani* and *Fusarium* spp. (http://www.epa.gov/opp00001/chem_search/reg_actions/registration/fs_PC-006493_13-Mar-03.pdf). However, little is known about the bacterial determinants responsible for conferring ISR and plant growth promotion activities.

Whole-genome sequencing was performed using an Illumina Genome Analyzer IIx at the National Instrumentation Center for Environmental Management (Seoul, Korea), according to the manufacturer’s protocols. 2 × 101 bp reads, totaling 32,487,788 reads (3.28 Gb), were produced and *de novo* assembled using CLC Genomics Workbench version 4.8 after quality trimming and filtering (about 749-fold coverage after pretreatment). Sequence Read Archive (SRA) data are available under the accession no. SRS542401. The final assembly, 3,681,709 bp in length, consists of 55 contigs, with an *N*_50_ of 147,762 bp. The maximum contig size is 394,228 bp. The gene prediction and functional annotation were performed using the RAST server (6), which predicted 74 tRNAs and 3,859 coding sequences. The G+C content was 41.3%. According to the RAST analysis result, *Bacillus pumilus* SARF-032 was recognized as the closest neighbor to the strain INR7. The average nucleotide identity value between INR7 and SARF-32 strains, obtained by the JSpecies program (7), was only 88.2%, which implies that accurate species identification is required for INR7.

Genome analysis revealed that INR7 genome contains three non-ribosomal peptide synthetase gene clusters for the production of antibiotics such as surfactin, bacillibactin, and bacilysin. The genome contains biosynthesis genes for production of indole-3-acetaldehyde and 2,3-butanediol corresponding to elicitation of plant growth promotion and ISR (8). No homologous genes for levan sucrase or phytase were found in the genome, but other genes related to swarming and biofilm formation are well conserved. The results explain why the strain INR7 has been commercialized to elicit both ISR and plant growth promotion. The genome information may be useful to improve the strain as a biocontrol agent.

### Nucleotide sequence accession numbers.

This whole-genome shotgun project has been deposited at DDBJ/EMBL/GenBank under the accession no. AYTK00000000. The version described in this paper is version AYTK01000000.
